# N-Acetyl-L-Cysteine Ameliorates BPAF-Induced Porcine Sertoli Cell Apoptosis and Cell Cycle Arrest via Inhibiting the ROS Level

**DOI:** 10.3390/toxics11110923

**Published:** 2023-11-11

**Authors:** Yue Feng, Junjing Wu, Runyu Lei, Yu Zhang, Mu Qiao, Jiawei Zhou, Zhong Xu, Zipeng Li, Hua Sun, Xianwen Peng, Shuqi Mei

**Affiliations:** 1 Hubei Key Laboratory of Animal Embryo and Molecular Breeding, Institute of Animal Husbandry and Veterinary, Hubei Academy of Agricultural Sciences, Wuhan 430064, China; fy914858517@163.com (Y.F.); wujujing@hbaas.com (J.W.); 13995765761@163.com (R.L.); zhangyu@hbaas.com (Y.Z.); qiaomu@hbaas.com (M.Q.); zhoujiawei@hbaas.com (J.Z.); xuzhong@hbaas.com (Z.X.); lizipeng@hbaas.com (Z.L.); sunhua@hbaas.com (H.S.); 2 College of Animal Science and Technology, Huazhong Agricultural University, Wuhan 430070, China; 3 Hubei Hongshan Laboratory, Wuhan 430070, China

**Keywords:** bisphenol AF, sertoli cell, apoptosis, cell cycle, N-acetyl-L-cysteine, ROS

## Abstract

Bisphenol AF (BPAF) is a newly identified contaminant in the environment that has been linked to impairment of the male reproductive system. However, only a few studies have systematically studied the mechanisms underlying BPAF-induced toxicity in testicular Sertoli cells. Hence, this study primarily aims to explore the toxic mechanism of BPAF on the porcine Sertoli cell line (ST cells). The effects of various concentrations of BPAF on ST cell viability and cytotoxicity were evaluated using the Counting Kit-8 (CCK-8) assay. The results demonstrated that exposure to a high concentration of BPAF (above 50 μM) significantly inhibited ST cell viability due to marked cytotoxicity. Flow cytometry analysis further confirmed that BPAF facilitated apoptosis and induced cell cycle arrest in the G2/M phase. Moreover, BPAF exposure upregulated the expression of pro-apoptotic markers BAD and BAX while downregulating anti-apoptotic and cell proliferation markers BCL-2, PCNA, CDK2, and CDK4. BPAF exposure also resulted in elevated intracellular levels of reactive oxygen species (ROS) and malondialdehyde (MDA), alongside reduced activities of the antioxidants glutathione (GSH), catalase (CAT), and superoxide dismutase (SOD). Furthermore, the ROS scavenger N-acetyl-L-cysteine (NAC) effectively blocked BPAF-triggered apoptosis and cell cycle arrest. Therefore, this study suggests that BPAF induces apoptosis and cell cycle arrest in ST cells by activating ROS-mediated pathways. These findings enhance our understanding of BPAF’s role in male reproductive toxicity and provide a foundation for future toxicological assessments.

## 1. Introduction

Bisphenol AF (BPAF) is a fluorinated compound widely used in various products, including food packaging, plastics, and pharmaceutical intermediates [1,2,3]. BPAF, released into the environment during production and usage, poses potential risks to ecosystems, human, and animal health [4,5,6]. In the past few years, BPAF has been identified not only in environmental media, water, and foodstuffs [7,8,9] but also in human body fluids and adipose tissue [10,11]. Studies suggest a significant association between exposure to bisphenol analogs and certain cases of infertility, particularly those of unknown etiology [12,13,14]. For example, exposure to BPAF has been linked to follicular development disorders in female animals, including premature ovarian failure (POF), ovarian cysts (OC), and polycystic ovary syndrome (PCOS) [15,16,17]. Bisphenol A (BPA) has been shown to disrupt testicular development in male animals, leading to reproductive toxicity [18]. Considering the structural similarities between BPAF and BPA, it is reasonable to hypothesize that BPAF could exhibit similar reproductive toxicity. Research in recent years has demonstrated that BPAF exposure can trigger follicular atresia through apoptosis of granulosa cells [19]. Moreover, BPAF exposure has been observed to disrupt sex hormone levels and vitellogenin expression in zebrafish, indicating endocrine-disrupting effects [20]. However, the precise mechanism by which BPAF affects male reproductive dysfunction, particularly the functionality of porcine testis Sertoli cells, remains poorly understood. Elucidating the precise mechanisms underlying BPAF’s impact on male reproductive function is essential for assessing and mitigating its potential risks to reproductive health.

In the mammalian testis, Sertoli cells serve crucial functions in regulating male germ cell development (spermatogenesis) by providing essential nutritional and structural support [21]. Adjacent Sertoli cells form the blood-testis barrier (BTB), a critical structure that prevents harmful substances from penetrating the seminiferous tubules, thereby safeguarding germ cells [22]. The specialized microenvironment maintained by the BTB is fundamental to ensuring the proper conditions for spermatogenesis. Swine testicular (ST) cells, representing immature Sertoli cells, exhibit a rapid proliferative capacity. The population of these immature cells influences the subsequent development of mature Sertoli cells, ultimately affecting sperm production and testis size [23,24,25]. Thus, the efficiency of spermatogenesis is closely tied to the proliferation and functionality of immature Sertoli cells [26,27]. Therefore, Sertoli cell damage is an important indicator of male reproductive impairment. Investigating the impact of BPAF on Sertoli cells offers vital insights into the compound’s harmful effects on male reproductive health, enhancing our understanding of its broader ecological and public health implications.

Reactive oxygen species (ROS) are reactive molecules or ions characterized by high oxidative activity. Excessive ROS production overwhelms cellular antioxidant defenses, resulting in oxidative damage, cellular apoptosis, and cell cycle arrest [28,29]. Various toxic substances, including endocrine-disrupting chemicals (EDs) such as BPAF, commonly induce oxidative stress, damaging Sertoli cells [19,30]. N-acetyl-L-cysteine (NAC) acts as an antioxidant and a glutathione (GSH) precursor, which safeguards cells from oxidative damage caused by ROS. However, whether BPAF induces Sertoli cell apoptosis and cell cycle arrest through oxidative stress mechanisms remains unclear. Thus, the aim of this investigation was to assess whether BPAF induces oxidative stress in Sertoli cells and to determine its impact on Sertoli cell apoptosis and cell cycle arrest. These findings will provide insights into the potential molecular pathway of BPAF in male reproductive dysfunction. This study, therefore, aimed to determine if BPAF triggers oxidative stress in Sertoli cells, subsequently influencing apoptosis and cell cycle dynamics. Understanding these processes could reveal key molecular pathways influenced by BPAF, shedding light on its role in male reproductive dysfunction.

## 2. Materials and Methods

### 2.1. Reagents

Bisphenol AF (BPAF; molecular weight (MW): 336.23 g/mol; ≥99% purity), dihydroethidium (DHE; MW: 315.41 g/mol; ≥95% purity), and N-acetyl-L-cysteine (NAC; MW: 163.19 g/mol; ≥99% purity) were sourced from Sigma-Aldrich (St. Louis, MO, USA). The Cell Counting Kit-8 (CCK-8), Annexin V-FITC/PI Apoptosis Detection Kit, and Cell Cycle Detection Kit were procured from Nanjing KeyGen Biotech (Nanjing, China). The glutathione (GSH) assay kit, superoxide dismutase (SOD) assay kit, catalase (CAT) assay kit, and malondialdehyde (MDA) assay kit were purchased from Nanjing Jiancheng Bioengineering Institute (Nanjing, China).

### 2.2. Cell Culture and Treatment

The swine testicular (ST) cell line (ATCC Cat# CRL-1746, RRID: CVCL_2204) was identified as immature testicular Sertoli cells, isolated from the fetal testes of 80- to 90-day-old swine [31] and procured from the Cell Bank of Wuhan University (Wuhan, China). ST cells were maintained at 37 °C with 5% CO_2_ in DMEM/High Glucose Medium (HyClone, Logan, UT, USA) supplemented with 10% fetal bovine serum (FBS) (Gibco, Grand Island, NY, USA). BPAF was prepared as a stock solution in dimethyl sulfoxide (DMSO) and stored at −20 °C. The stock solution was subsequently diluted with the culture medium to attain various concentrations of BPAF. The indicated concentrations of BPAF were applied to ST cells. To conduct the NAC rescue experiment, ST cells were pretreated with 5 mM NAC for 2 h before BPAF exposure [30]. Subsequently, we set up the three different co-treatment groups: 0 μM BPAF + 0 mM NAC group (CON); 50 μM BPAF + 0 mM NAC group (BG); 50 μM BPAF + 5 mM NAC group (NBG).

### 2.3. CCK-8 Assay

For the CCK-8 assay, ST cells were seeded into 96-well plates at 1 × 10^4^ cells/well density. Following overnight incubation at 37 °C with 5% CO_2_, ST cells were exposed to various concentrations of BPAF (0 μM, 1 μM, 10 μM, 25 μM, 50 μM, 75 μM, and 100 μM) for 24, 48, or 72 h, respectively. After the specified incubation period, we added 10 microliters of CCK-8 reagent to each well and incubated them at 37 °C for another 2 h. The optical density was then measured at 450 nm using a microplate reader (Bio-Rad, Hercules, CA, USA).

### 2.4. Cell Apoptosis Assays

The level of cell apoptosis was analyzed by utilizing the Annexin V-FITC/PI apoptosis detection kit. ST cells, seeded in 6-well plates at a density of 1 × 10^6^ cells/well, were cultured overnight before exposure to the designated treatments. ST cells were then harvested by centrifuging at 500× *g* for 10 min at 4 °C, followed by three washes with cold PBS. The cells were stained in the dark using Annexin V-FITC and PI for 10 min. Each sample was mixed with 400 µL of 1× binding buffer to facilitate detection. The apoptosis rate was then assessed using FACS Calibur Flow Cytometry (Beckman Coulter, Brea, CA, USA).

### 2.5. Cell Cycle Analysis

To assess the effects of BPAF exposure on the cell cycle, the cell cycle detection kit was employed following the manufacturer’s instructions. ST cells were seeded into 6-well plates at a density of 1 × 10^6^ cells/well and cultured overnight. ST cells were first transferred to flow cytometry tubes and centrifuged at 1500 rpm for 5 min, resulting in the formation of cell pellets. The cells were then washed with PBS and fixed in cold 70% ethanol overnight at 4 °C. Post-fixation, the cells were washed again with cold PBS. The cells were then incubated in 100 µL of PI staining solution at 37 °C for 10 min. Using a Flow Cytometer (Beckman Coulter, USA), the distribution of cells across the various phases of the cell cycle was analyzed.

### 2.6. Western Blot

ST cells were placed into 6-well plates at 1 × 10^6^ cells/well density and cultured overnight for western blot analysis. ST cells were subjected to lysis using a cell lysis buffer containing 1% phenylmethanesulfonyl fluoride (Biyotime, Nantong, China) for 20 min on ice. The resulting lysates were then subjected to centrifugation at 12,000 rpm for 10 min to obtain the supernatant. The protein concentration of the lysate was measured with the BCA protein detection kit (Beyotime, Nantong, China). The cellular proteins were denatured by mixing them with 5× loading buffer and boiling them for 10 min. The protein samples were first separated using SDS-PAGE with an initial voltage of 80 V for 30 min, followed by 120 V for 80 min. Subsequently, the protein samples were transferred to a PVDF membrane (Millipore, Burlington, MA, USA) at a constant current of 200 mA for 90 min. We blocked non-specific binding sites on the proteins with 5% nonfat milk for 2 h, followed by overnight incubation at 4 °C with the primary antibodies. After washing the membranes, we incubated them with secondary antibodies for 2 h at room temperature. Immunoreactive bands were visualized utilizing the Clarity Western ECL Substrate Kit (Bio-rad, USA). The Image Quant LAS4000 system (GE Healthcare Life Sciences, Marlborough, MA, USA) was employed to capture the images. The primary antibodies used were BAX (A0207, ABclonal; 1:1000), BCL-2 (AF6139, Affinity; 1:1000), BAD (A19595, ABclonal; 1:1000), PCNA (A12427, ABclonal; 1:800), CDK2 (A0094, ABclonal; 1:800), CDK4 (A11136, ABclonal; 1:1000), and β-actin (AC028, ABclonal; 1:20,000). The second antibody was HRP Goat Anti-Rabbit IgG (AS014, ABclonal; 1:5000). Immunoblotting for β-actin was performed as a protein loading control.

### 2.7. Real-Time Quantitative PCR (RT-qPCR)

Cells (1 × 10^6^ cells/well) were seeded in a 6-well plate and incubated for 24 h. Total RNA extraction from ST cells was performed using the TRIzol™ Reagent (Invitrogen, Waltham, MA, USA), followed by isopropanol precipitation and chloroform extraction. Subsequently, the RNA underwent two washes with 75% ethanol. A gDNA eraser kit (Takara, Kusatsu, Japan) was employed to eliminate genomic DNA. RNA to cDNA was converted using a reverse transcription kit (Takara, Japan), preparing the samples for subsequent RT-qPCR analysis. RT-qPCR analysis was performed on a CFX384 Real-Time PCR Detection System (Bio-Rad, USA) utilizing the SYBR Green Supermix (Bio-Rad, USA). All of the target gene transcripts in each sample were detected three times. The 2^−∆∆Ct^ method was employed to calculate the relative mRNA expression of genes, with β-actin being used as the housekeeper gene. The primer sequences used in this study can be found in Table 1.

### 2.8. Detection of Intracellular ROS

ST cells were seeded into 6-well plates at 1 × 10^6^ cells/well density, cultured overnight, and then subjected to the respective treatments. The DHE, a fluorogenic probe, was performed to assess ROS levels. The cells were subjected to three cycles of PBS washing, each lasting 10 min, followed by a 30-min incubation with 10 µM DHE in PBS at 37 °C while shielded from light. The fluorescence was examined and captured utilizing a fluorescence microscope (Zeiss, Oberkochen, Germany), with excitation at 518 nm and emission at 605 nm. The fluorescence intensity of DHE-labeled positive staining was quantified with Image J software (version 2.0) in four randomly selected fields for each group to compare the levels of ROS production. The mean fluorescence intensity of the control group was counted as 1 to normalize the fluorescence intensity of the treatment group.

### 2.9. Oxidative Stress-Related Molecular Assays

ST cells were plated at 1 × 10^6^ cells/well in 6-well plates. The ST cells in PBS were homogenized on ice and centrifuged to obtain the supernatants for subsequent biochemical assays. Bovine serum albumin (BSA) was employed as the standard for measuring protein concentration. The GSH assay kit (colorimetric method), MDA assay kit (TBA method), SOD assay kit (WST-1 method), and CAT assay kit (visible light method) were used to detect intracellular levels of GSH, MDA, SOD, and CAT, respectively. The manufacturer’s instructions were strictly adhered to during all procedures. A microplate reader was employed to measure the levels, which were subsequently normalized to the protein content.

### 2.10. Statistical Analysis

All experiments were conducted in triplicate and repeated three times to validate the reliability of the results. The data were presented as the mean ± standard deviation (SD). Statistical analysis involved using a two-tailed Student’s *t*-test to compare the experimental and control groups. *p* < 0.05 was deemed statistically significant.

## 3. Results

### 3.1. BPAF-Inhibited Porcine ST Cell Viability

**Figure 1:**
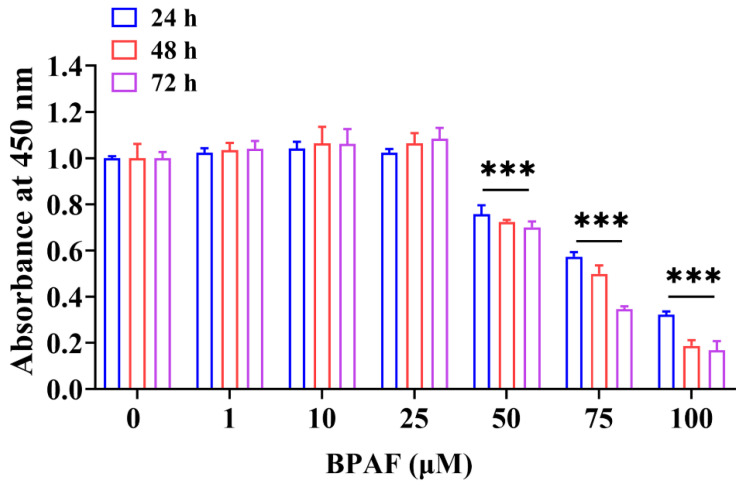
Effect of Bisphenol AF (BPAF) on swine testicular (ST) cell viability. The cell viability was assessed through the CCK-8 assay after exposing the ST cells to various concentrations (0 μM, 1 μM, 10 μM, 25 μM, 50 μM, 75 μM, and 100 μM) of BPAF for 24, 48, and 72 h. The data presented here represent the mean ± SD of at least three separate experiments. *** *p* < 0.001 versus 0 μM.

To evaluate BPAF’s impact initially, we assessed the cytotoxicity across a range of concentrations from 0 to 100 μM on ST cell viability. This assessment was conducted via the CCK-8 assay, exposing the cells to BPAF for 24, 48, and 72 h. Our results showed a significant decrease in cell viability at BPAF concentrations of 50 μM and above, contrasting with low concentrations that surprisingly increased cell viability (Figure 1). Consistently, similar results have already been reported in MCF-7 cells [30,32]. Since 50 μM BPAF was the lowest concentration capable of reducing ST cell viability to ~75% compared with the control group (0 μM), this concentration was selected for subsequent studies.

### 3.2. BPAF Induced Cell Apoptosis and Cell Cycle Arrest at G2/M Phase of Porcine ST Cells

**Figure 2:**
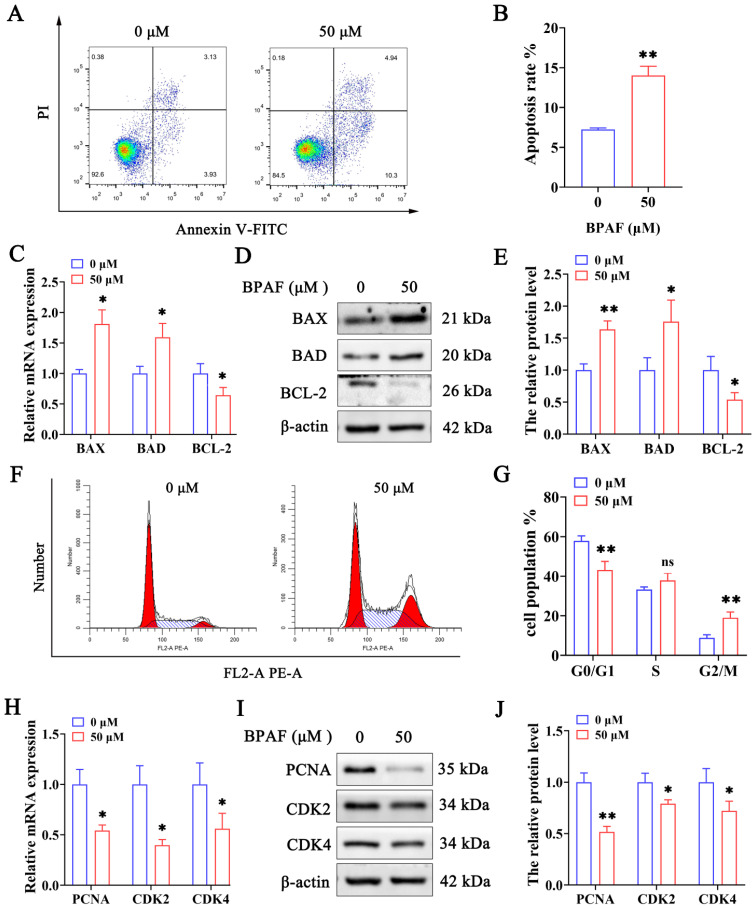
Effects of BPAF on apoptosis and cell cycle in ST cells. (**A**) Flow cytometry analysis of ST cell apoptosis using Annexin V-FITC/PI staining following 50 μM BPAF exposure (**B**) Quantification of apoptosis rates in ST cells after 50 μM BPAF treatment. (**C**,**D**) The expression levels of BAX, BAD, and BCL-2 were determined using RT-qPCR (**C**) and western blot (**D**) after 50 μM BPAF treatment. (**E**) The western blot quantification is shown as a bar graph. (**F**) Flow cytometry was utilized to determine the relative proportion of cells with each cell cycle phase after 50 μM BPAF treatment. (**G**) Histogram and statistical analysis for cell cycle distribution in ST cells. (**H**,**I**) The expression levels of PCNA, CDK2, and CDK4 were determined through RT-qPCR (**H**) and western blot (**I**) after 50 μM BPAF treatment. (**J**) The western blot quantification is shown as a bar graph. The data presented here represent the mean ± SD of at least three separate experiments. * *p* < 0.05 and ** *p* < 0.01 versus 0 μM. ns, not significant (*p* > 0.05).

To investigate the role of apoptosis in BPAF-induced cytotoxicity in ST cells, we employed the Annexin V-FITC/PI double staining assay. Figure 2A,B show a significant increase in apoptosis rate in ST cells treated with 50 μM BPAF compared to the control group (0 μM). To elucidate the mechanisms of BPAF-induced cell apoptosis, the mRNA and protein levels of apoptosis markers were measured via RT-qPCR and western blot analysis. As expected, BPAF treatment (50 μM) significantly upregulated BAX and BAD mRNA and protein levels while downregulating BCL-2 expression (Figure 2C–E). These findings firmly establish that BPAF triggers apoptosis in ST cells. 

We performed flow cytometry analysis to investigate the impact of BPAF on cell cycle distribution. Figure 2F,G illustrate that BPAF treatment increased the proportion of ST cells in the G2/M phase while significantly reducing those in the G0/G1 phase. To elucidate the mechanisms of BPAF-triggered cell cycle arrest, the expression levels of cell cycle-related genes were assessed using RT-qPCR and western blot analysis. BPAF treatment (50 μM) reduced PCNA, CDK2, and CDK4 levels (Figure 2H–J). These findings suggest that BPAF triggers cell cycle arrest in ST cells. These results are in strong agreement with previous studies [33,34].

### 3.3. BPAF-Triggered Oxidative Stress in Porcine ST Cells

**Figure 3:**
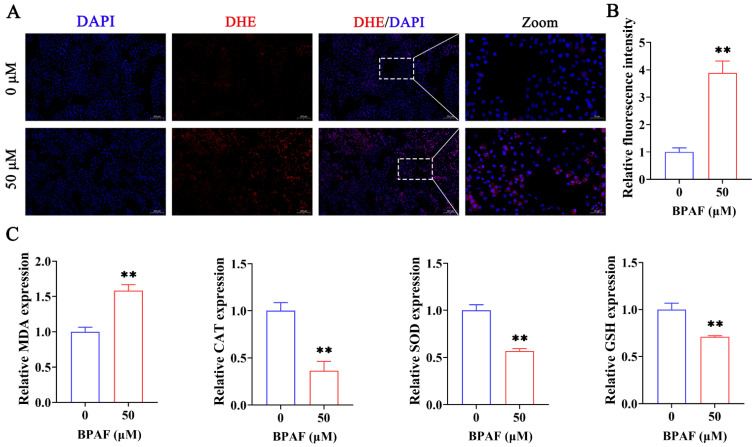
Effects of BPAF on intracellular ROS and oxidative stress indicators in ST cells. (**A**,**B**) Representative images of DHE staining and quantitative analysis of image fluorescence intensity after treatment with a concentration of 50 μM BPAF indicate increased ROS levels. (**C**) Measurements of oxidative stress markers, including MDA, CAT, SOD, and GSH, reveal an oxidative imbalance post-BPAF exposure. The data presented here represent the mean ± SD of at least three separate experiments. ** *p* < 0.01 versus 0 μM.

It is well established that excessive ROS generation triggers cellular responses, including cell cycle arrest and apoptosis [28,29]. To explore this, we used DHE staining to assess intracellular ROS production following treatment with 50 μM BPAF. Figure 3A,B demonstrate that BPAF treatment elevated ROS levels in porcine ST cells. MDA, CAT, SOD, and GSH are pivotal markers in evaluating oxidative stress, serving as indicators of oxidative injury [35,36]. Therefore, these markers were monitored to ascertain BPAF’s impact on oxidative stress in ST cells. Our oxidative stress assay revealed that BPAF exposure increased MDA levels and decreased CAT, SOD, and GSH activities (Figure 3C). These results indicate that BPAF upregulated the ROS level and induced oxidative stress in ST cells.

### 3.4. NAC Relieves Cell Viability and Oxidative Stress in BPAF-Induced Porcine ST Cells

**Figure 4:**
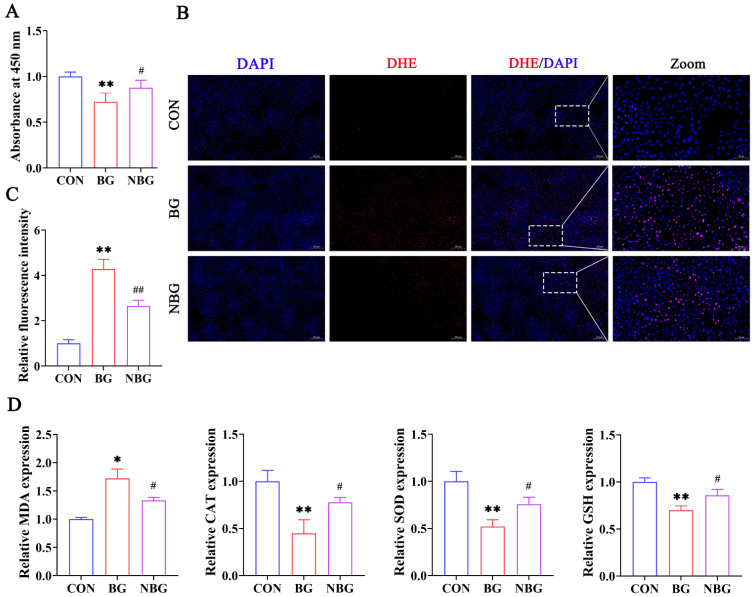
NAC mitigates BPAF-induced cytotoxicity and oxidative stress in ST cells. (**A**) Cell viability was assessed via the CCK-8 assay in ST cells across three treatment groups: CON, BG, and NBG. (**B**,**C**) Representative images of DHE staining and corresponding quantitative fluorescence analysis depict ROS levels in the three groups. (**D**) Comparative analysis of oxidative stress markers (MDA, CAT, SOD, and GSH) post-treatment, highlighting NAC’s protective role against BPAF-induced oxidative alterations. 0 μM BPAF + 0 mM NAC group, CON; 50 μM BPAF + 0 mM NAC group, BG; 50 μM BPAF + 5 mM NAC group, NBG. The data presented here represent the mean ± SD of at least three separate experiments. * *p* < 0.05 and ** *p* < 0.01 versus the CON. ^#^ *p* < 0.05 and ^##^ *p* < 0.01 versus the BG.

To validate ROS’s role in BPAF-induced cytotoxicity, we examined how NAC, an antioxidant, affects ST cell viability. CCK-8 results showed a significant decrease in ST cell viability post-BPAF (50 μM) treatment, which was mitigated by a 2-h pretreatment with NAC (5 mM) (Figure 4A). These findings confirm NAC’s capability to ameliorate the inhibitory effect of BPAF on ST cell viability.

We then measured ROS levels and oxidative stress indicators across three treatment groups: CON, BG, and NBG. Compared to the CON group (0 μM BPAF + 0 mM NAC group), the BG group (50 μM BPAF + 0 mM NAC group) exhibited notably higher ROS and MDA levels and significantly reduced SOD, GSH, and CAT activities (Figure 4B–D). In contrast, the NBG group (50 μM BPAF + 5 mM NAC group) displayed significantly reduced ROS and MDA levels and enhanced SOD, GSH, and CAT activities compared to the BG group (Figure 4B–D). Thus, NAC effectively counteracts BPAF-induced intracellular ROS production and oxidative stress in ST cells, highlighting its protective role.

### 3.5. NAC Alleviates BPAF-Triggered Apoptosis and Cell Cycle Arrest in Porcine ST Cells

**Figure 5:**
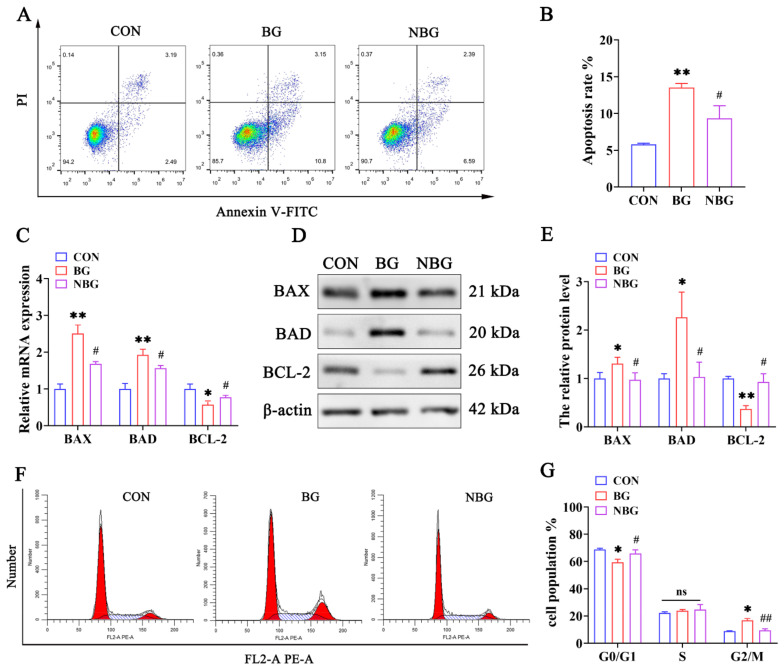

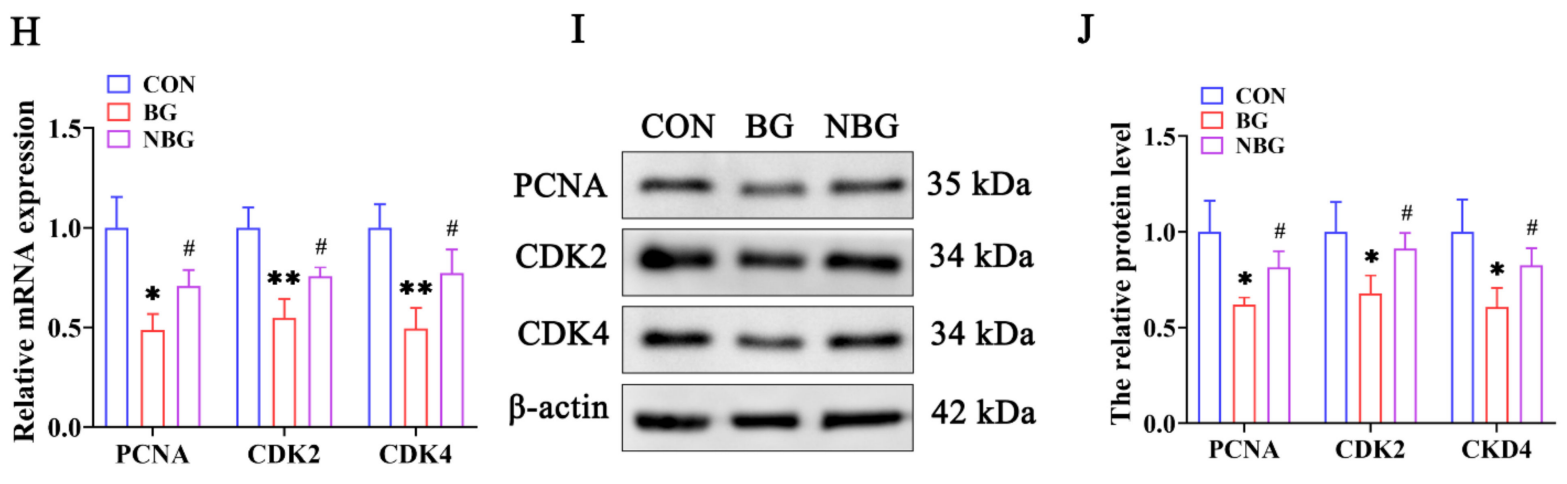
NAC attenuates BPAF-induced apoptosis and cell cycle arrest in ST cells. (**A**) Apoptotic rates in ST cells were analyzed via Annexin V-FITC/PI staining and flow cytometry post-treatment in the CON, BG, and NBG groups. (**B**) Quantitative assessment of apoptosis following the treatments. (**C**,**D**) The expression levels of BAX, BAD, and BCL-2 were determined using RT-qPCR (**C**) and western blot (**D**) across the treatment groups. (**E**) Quantitative representation of the western blot results in bar graph format. (**F**) Cell cycle phase distribution post-treatment, determined through flow cytometry, indicating shifts in cell cycle dynamics. (**G**) Histogram and statistical analysis illustrating the cell cycle alterations. (**H**,**I**) The expression levels of PCNA, CDK2, and CDK4 were determined using RT-qPCR (**H**) and western blot (**I**) across the treatment groups. (**J**) Quantitative representation of the western blot results in a bar graph format. 0 μM BPAF + 0 mM NAC group, CON; 50 μM BPAF + 0 mM NAC group, BG; 50 μM BPAF + 5 mM NAC group, NBG. The data presented here represent the mean ± SD of at least three separate experiments. * *p* < 0.05 and ** *p* < 0.01 versus the CON. ^#^ *p* < 0.05 and ^##^ *p* < 0.01 versus the BG. ns, not significant (*p* > 0.05).

To determine ROS’s role in BPAF-triggered apoptosis and cell cycle arrest, ST cells were pretreated with NAC for 2 h prior to treatment with 50 μM BPAF. Figure 5A,B demonstrate that NAC pretreatment effectively reduced BPAF-induced apoptosis. Furthermore, the mRNA and protein levels of apoptosis markers were assessed via RT-qPCR and western blot analysis. We observed that BAX and BAD mRNA and protein levels increased significantly in the BG group compared to the CON group, yet they decreased significantly in the NBG group relative to the BG group. Conversely, BCL-2 levels followed an opposite trend (Figure 5C–E). As expected, NAC pretreatment partially reversed the BPAF-induced cell cycle arrest (Figure 5F,G). In the NBG group, PCNA, CDK2, and CDK4 levels were significantly elevated compared to the BG group, which, in contrast, exhibited lower levels than the CON group (Figure 5H–J). These findings confirm that ROS accumulation and oxidative stress are crucial drivers of BPAF-induced apoptosis and cell cycle arrest in ST cells.

## 4. Discussion

Sertoli cells, crucial for testis development and spermatogenesis, provide essential support, nourishment, and immunological protection to developing germ cells [37,38]. Given their role as primary targets for environmental and chemical toxicants, Sertoli cells have gained wide use in investigations of the cytotoxicity of male reproductive dysfunctions [39,40]. Consequently, dysfunction in Sertoli cells is implicated in various male subfertility and infertility disorders [41,42,43]. Exposure to environmental chemical mixtures likely perpetuates these dysfunctions [44,45]. Increasing evidence now links ROS accumulation and redox imbalance in the testis to male reproductive dysfunctions induced by environmental pollutants and chemical toxicants [46,47].

BPAF is regarded as a primary alternative to BPA. Although BPAF has been found in water, food, and environmental media [7,48,49], little is known about its impact on male reproductive function, especially testicular Sertoli cells. This research aims to explore the potential BPAF cytotoxicity on Sertoli cells’ growth and to determine the underlying molecular mechanism. Wu et al. [50] demonstrated that in vitro treatment with a 50 μM concentration of BPAF disrupts Sertoli cells’ cytoskeleton and compromises their tight junction permeability. In vivo treatment of mice with 50 mg/kg/d BPAF also impaired spermatogenesis [50]. Therefore, we believe there may be a correlation between in vitro cytotoxicity assays and in vivo toxicity. Nonetheless, conclusive interpretations necessitate further in vivo experimental investigations. BPAF exhibits dual effects on cell proliferation, depending on its different concentrations [30,32]. Growing evidence suggests that while low doses of BPAF can enhance cell viability, higher doses impede cellular functions, triggering apoptosis, autophagy, and cell cycle arrest [19,51]. Consistent with previous studies, a low concentration of BPAF in our study could increase ST cell viability. However, when the concentration of BPAF exceeded 50 μM, it inhibited ST cell viability, induced ST cell apoptosis and cell cycle arrest, and significantly enhanced the toxic effect on cells. Moreover, Yin L et al. [52] reported increased DNA damage and synthesis in Sertoli cells exposed to low BPAF doses. The above results suggest that Sertoli cells may be highly sensitive to BPAF toxicity and that BPAF may have profound long-term effects on the reproductive system. Notably, prior studies indicated that BPA concentrations of up to 400 μM are necessary to compromise Sertoli cell viability and induce apoptosis, suggesting a higher sensitivity of these cells to BPAF-induced toxicity than BPA.

Maintaining moderate ROS levels is essential for redox homeostasis and normal cellular function [53]. However, excessive ROS can overwhelm cellular antioxidant capacity, initiating abnormal cell death. Several markers, including GSH, CAT, SOD, and MDA, serve as indicators for assessing the body’s oxidative status. SOD, an antioxidant enzyme, maintains the balance of oxidation/anti-oxidation during oxidative stress [54]. CAT, a scavenging enzyme, decomposes hydrogen peroxide into water and oxygen and is crucial for eliminating peroxisome-generated ROS [55]. GSH acts as an intracellular antioxidant, safeguarding cells against oxidative stress [56]. MDA, a byproduct of oxidative damage, serves as a significant oxidative stress marker [57]. The levels of oxidative stress indicators can reflect the severity of oxidative stress damage. Oxidative stress is the primary mechanism of cell damage induced by exogenous chemicals. BPAF exposure causes oxidative stress in human red blood cells through elevated ROS and MDA levels and diminished SOD and CAT activities [58]. Our findings align with these results, showing BPAF-induced ROS and MDA levels increase, coupled with decreased SOD, CAT, and GSH activity. These findings demonstrate that porcine ST cells experienced oxidative damage and that oxidative stress was crucial to the cytotoxicity induced by BPAF. Therefore, we hypothesize that BPAF damages ST cells by amplifying oxidative stress and diminishing their antioxidant capacity. 

A widely used antioxidant, NAC, directly stimulates cellular GSH synthesis and curtails ROS production [59]. Accordingly, NAC mitigates impairment in the nonylphenol-exposed mouse Sertoli cell line (TM4) by reducing ROS production [60]. This study utilized optimal NAC treatment concentrations and durations based on established literature [30,61,62,63]. 5 mM NAC alleviated BPAF-induced ROS accumulation, MDA enhancement, and reduced GSH, SOD, and CAT in ST cells. This outcome underscores NAC’s efficacy in mitigating BPAF-induced oxidative stress. Furthermore, NAC alleviated BPAF’s adverse effects on ST cell activity, apoptosis, and cell cycle progression. These results suggest that NAC counteracts BPAF-triggered cytotoxicity by modulating oxidative stress-related apoptosis and cell cycle arrest. These findings are consistent with several previous studies [64,65,66]. In vitro studies corroborate that various natural antioxidants, including NAC, preserve Sertoli cell function by mitigating ROS accumulation [67,68,69]. However, in vivo studies are necessary to delineate NAC’s mitigating effects on BPAF-induced toxicity precisely.

## 5. Conclusions

In conclusion, our findings demonstrate that BPAF exposure causes ST cell apoptosis and cell cycle arrest by promoting the production of ROS. Moreover, the protective effect of the antioxidant NAC against BPAF-triggered cytotoxicity in ST cells was demonstrated. Therefore, improving cellular antioxidant defense mechanisms may offer a therapeutic strategy to mitigate BPAF-induced dysfunction in Sertoli cells. This study significantly advances our understanding of BPAF’s reproductive toxicity, serving as a critical reference for future toxicological studies on BPAF’s impact on male reproductive health. 

## Figures and Tables

**Table 1:** Sequences of the primers.

Gene	The Sequence of the Primers
BAX	F: GCCGAAATGTTTGCTGACG R: CAGCCGATCTCGAAGGAAG
BAD	F: CAAAGGCCGATTCCCTTCCT R: GGCGGCGTTAGGGTTAATCT
BCL-2	F: TCCAGGCAGTTTAATACATTC R: TCCCTTTATACACTGGGTGA
PCNA	F: ACCGCTGCGACCGCAATTTG R: ACGTGCAAATTCACCAGAAGGCATC
CDK4	F: GCGGAGATTGGTGTTGGTG R: CATTGGGGACTCTTACGCTCTT
CDK2	F: GTGGCTGCATCACAAGGAGG R: CCGGAAGAGCTGGTCAATCT
β-actin	F: CCAGGTCATCACCATCGG R: CCGTGTTGGCGTAGAGGT

## Data Availability

The data that support the findings of this study are available from the corresponding author upon reasonable request.
